# Working with Cambridge physiologists

**DOI:** 10.1098/rsnr.2007.0036

**Published:** 2008-01-09

**Authors:** E.M. Tansey

**Affiliations:** Wellcome Trust Centre for the History of Medicine, University College London183 Euston Road, London NW1 2BE, UK

Clive Hood, a technician in the Physiological Laboratory, Cambridge, was interviewed in April 1995 by Dr E. M. Tansey and Dr Ann Silver, the interview being transcribed and edited by Tansey. Starting his career with the first Lord Adrian FRS, Clive also worked with Professor William Rushton FRS and Professor Fergus Campbell FRS. This is an edited extract of that interview, the recording of which was supported financially by the Physiological Society. The original tape and transcript will be deposited in the Physiological Society Archives, Archives and Manuscripts, The Wellcome Library, London.

Tansey: Tell me how you got started, Clive.

Hood: I left grammar school on the 23rd of July 1947, which was a Tuesday, and I actually started in physiology the very next day in the experimental classroom for medical students and actually I was employed by Lord Adrian; he interviewed me.^1^ I think he must have read my CV upside down, or else I am sure I wouldn't have got the job.

Tansey: Had you always been interested in science at school?

Hood: Oh yes, I liked science. I mean the classics and that were out of the window as far as I was concerned. I always thought it was a waste of time—and history, I mean history, you learn history so you can go to university to learn history to go back to school to teach people history, and it's just a vicious circle. But sciences yes.

Tansey: Did you try any other labs or did you particularly want to come to physiology?

Hood: I just happened to see the advertisement in the *Cambridge Evening News* and came along and got it and been here ever since.^2^

Tansey: E. D. Adrian employed you as his technician?

Hood: No, no. As a technician in the experimental classroom. The person up there in charge was a Mr William Pawley; he was a great technician. He was really good at mammalian stuff and nerves and everything. That was when the classes weren't so great in number of course. They have escalated appreciably since then.

Tansey: So when you started, and there was Mr Pawley, did he sort of teach you the tricks of the trade?

Hood: Yes, the tricks of the trade. I also learnt a lot from a Mr Cook, who was Hodgkin's technician.^3^ Because I had never done any machine work or instrument-making, and so I learnt most of the stuff from him. He had a lovely workshop which I could utilize.

Tansey: So you also learnt all of the surgical skills as well, in the classroom, and then you had to do your National Service.

Hood: From Mr Pawley I learnt the surgical skills. That's right. As a matter of fact, I was only two weeks and five days in the RAF. They discharged me with a perforated eardrum.

Silver: You were also going to say a little bit about your standing in for Les Hatton.

Hood: Oh yes, this must have been in 1948. On the occasion Les Hatton, who was Lord Adrian's technician, was on holiday, I was allocated to assist Lord Adrian, *assist* in italics, because he was a sort of one-man band. The daily work used to be to put outside his door—some fully charged wet cells, batteries, some loaded hot-water bottles and a rabbit in a basket. You'd put that there and then you'd probably retire to the prep room to put the kettle on to fill more hot-water bottles and then you'd hear the door go and all the bits and pieces would disappear and much later that day the door would open and there was in a bucket the carcass of the rabbit for disposal. That was virtually the sum total of assisting Lord Adrian. He was so self-sufficient. In fact he would never allow people into his room.

Tansey: This was the famous basement room, was it?

Hood: It was in the basement and that room, you really had to see it to believe it. It was like the wedding banqueting room in Dickens's *Great Expectations*. Right opposite the door as you go in, there was this Keith Lucas gas mask from the First World War and the two cylindrical eyes would be staring at you through stalactites of matted cobwebs and it was incredible.^4^ Adrian would never allow anybody to touch anything. He was absolutely furious if anything was moved or touched, and all his tools and instruments of the trade, you could tell where they belonged, because they'd got the imprint everywhere of the dust. A pair of pliers, you'd know exactly where they'd go because of the dust. It was incredible.

Tansey: I didn't even know that he'd ever had a technician.

Hood: Well, he didn't, because he used to do everything himself.

Tansey: So what did this man Les do?

Hood: He used to do projection work as well, and had other duties, but his prime function was to make sure that those bits and pieces were there every day and disposed of. And, of course, the next day Les would come in and there outside his door would be the cold water bottles, discharged cells and the cat basket, which were ready for the next day. He'd ship them in.

Tansey: So how often did you have to ‘assist’ Adrian?

Hood: I'd perhaps have a week or so per session of this, in Les Hatton's absence. But there were two visitors from America and they wanted to come and see where the great Adrian worked, you see. So, knowing that he was away, I took them along to this room and opened the door, and these Americans immediately thought, oh he's just taking the mickey and nearly set about me. They just couldn't believe that the great Adrian could work in a room of that nature. And I do believe that he actually got a medal for hygiene of all things. He actually did get one.

Tansey: Wasn't there something that the room was flooded?

Hood: That was much, much later. In fact, I think that was the finish of him.

Tansey: I remember Richard Adrian telling me.^5^

Hood: Talking about Richard, I think it was only about a couple of weeks after Lord Adrian died I was talking to Richard and I said to him, ‘Oh by the way, what do we call you now?’ He thought a bit, and then said, ‘I shall still respond to “Hey, you” ’. He's a fantastic chap. He's got breeding right through him and I think he's a fabulous fellow.^6^

Tansey: How is he?

Hood: Not too good.

Tansey: He wrote me a lovely letter about the women physiologists book.^7^ A terribly nice one. [Going to lunch during this interview, we heard the sad news that Richard Adrian had died that morning.]

Silver: To go back to Les Hatton, you said that he often used to just be there with his kettles boiled in case Lord Adrian needed something.

Hood: That's right, and if anybody came along and looked in you'd see him twiddling the knobs on top of the accumulators or something like that—he's a busy man. But he had to waste time like that, because if something was called for he had to do it immediately for Lord Adrian.

Tansey: What other reminiscences do you have of him?

Hood: Lord Adrian?

Tansey: Did you know much about him?

Hood: No, I didn't know much about him; I didn't know he was an eminent man. I wasn't so brash or forward at the time, I was an innocent 16-year-old from grammar school. I don't honestly remember anything. He used to take the stairs about eight at a time. That was before he fell over a cliff with his wife and she lost a leg, didn't she? Because she used to be a magistrate, didn't she?^8^

Silver: Did he come down eight at a time? Because Andrew Huxley goes up two at a time and down two at a time. It's terrifying to see him.

Hood: Yes, well, three weeks ago I saw Andrew Huxley on the stairs so I said to him, ‘Oh, hello, how are you keeping lately?’ and he said, ‘It's not me I'm concerned about, it's my mother.’ Well, there's a man of over 80 and his mother was 104. As it so happened, she only died about three weeks after that conversation on the stairs.^9^

Silver: Yes, she died of pneumonia.

Tansey: Can we go back to your return after your weeks of National Service?

Hood: And on coming back here, that was when they put me with Rushton to help with his research on vision. He had just returned from Stockholm or somewhere, and was switching from nerve physiology to research on vision. That would have been in the beginning of 1950.^10^

Tansey: Did you still have to do general teaching duties or were you just Rushton's assistant?

Hood: Sometimes, yes. They would call on me to do decerebrations or something of that nature and ever since then I have been doing that. And dissection of crab nerves and that sort of thing. Sort of drag you away from research. Because I personally think that teaching is the prime function of a department, but most academics don't.

Tansey: So you had to learn a whole set of different skills, working with Rushton?

Hood: Oh yes, yes. Rushton was a person you didn't work with, you worked for.

Tansey: Yes, you made that point in the Fergus tribute book.^11^

Hood: Yes, that was quite an important point really, because Fergus you worked with and there was so much more of a rapport.

Tansey: Yes, well, he worked with you; he was ‘the Professor who worked with Clive’, wasn't he?

Hood: Yes, you remember the story; that was quite amusing. I don't know if you want me to tell you that now.

Tansey: Yes, please do.

Hood: Fergus left his expensive Paper Mate pen in the canteen and it was handed into the tea ladies and they were deliberating to whom it belonged, and one of the ladies said, ‘I know who that belongs to—that Professor who works with Clive’.^12^ When he heard of this, he was far more delighted than I was, I think, because he was such a marvellous man was Fergus.^13^

Tansey: Yes, I only met him a couple of times.

Hood: Yes, when you borrowed that capillary electrometer of Adrian's.^14^

Tansey: Yes, you pointed it out to me, didn't you? And the drawing.^15^

Silver: Was Rushton quite demanding to work with?

Hood: He was very, very demanding. Week after week we had normal people and colourblind people for recording from the back of the retina. He was trying to investigate the retinal organization of colourblind people and normals. It was very demanding, because we used to have a person helping us in the morning, in the afternoon, which necessitated putting atropine in their eye to blow up their pupil so it didn't constrict when strong light was shining.

Tansey: How did you get on with Rushton, then? You must have got on with him pretty well?

Hood: I did, I did. I think the word is I ‘handled’ him well.

Tansey: Did Rushton collaborate very much with other people?

Hood: Oh yes, yes. With Granit of course.^16^ That was what put him on the vision path, because he switched from nerves. I remember one eminent American vision scientist, Mathew Alpern, who actually came to Florida for the first year of Rushton's start there. He had quite an association with him scientifically.^17^

Tansey: And did you then help all the visitors and collaborators as well?

Hood: Oh yes, yes.

Silver: When you were in the States with Rushton you set up his lab for him, didn't you?

Hood: This is right. I was initially invited for the full year, but I had to cram a year's work in four months. That was quite a nice experience as a matter of fact.

Silver: But didn't you say that other people wanted you to set up labs for them, when they saw what you had done?

Tansey: When you say setting up a lab, I mean what did you have when you got there? Was it just an empty space? You had to get all the equipment?

Hood: Well, with Rushton we were lucky, because we had a forethought and ordered a terrific lot of stuff, apparatus-wise, and lenses, cameras, photocells; and that was already there waiting on our arrival. So we got the start there. But then we started from scratch actually building the Florida densitometer and we got that working, I should think, within the month.^18^ And everybody was told to go flat out on this project—all the services of the whole department—and they were also told to deal with me, rather than Rushton, knowing what Rushton was. And they all worshipped me [laughs], and they broke their backs to cooperate and things really got moving. In fact, the Director of the Institute called me in and he said, ‘How do you do it? Everybody's buzzing.’ Well, I said, ‘It's just personality, and money helps.’ And as a matter of fact, he gave quite a few of them a rise over there, just like that. Not like here, when you have to wait until the national increase and all that. Of course, they worshipped me even more then!

Tansey: What were you coming back to, as Rushton had moved?

Hood: Rushton moved. I knew that I was coming back to work with Fergus Campbell; he'd requested this and I knew exactly what I was coming back to. The same sort of research vision, but an entirely different sort of man to work for—well, work with.

Tansey: Did Rushton ever include your name in or on publications?

Hood: Oh, repeatedly. Oh yes. Only once did he ever put me as a co-author, but this didn't bother me at all, because I don't think I was entitled to be a co-author. He always used to put me in the acknowledgements, of course. And the only one I was mentioned [in] as a co-author was the Florida densitometer, believe it or not, which I had sort of had built and worked and everything.^19^

Tansey: How much of a contribution, if you can possibly assess it, do you think you made to Rushton's research?

Hood: Oh, a great deal. In fact I haven't got a copy of the letter, but it says in that letter that he wrote to me that I was to his research what his wife was to him in life.

Tansey: Absolutely critical, in fact?

Hood: Oh yeah, we did the writing together. I did all the figures and made sure that the points were large, so they all touched the theoretical line. Oh, I knew all the dodges at that time, I think.

Tansey: Did you pass on these dodges to other people? Were there other technicians and postdoctoral workers?

Hood: Oh yes, oh yes.

Tansey: What about students or Fellows? You probably trained quite a few, didn't you?

Hood: I wouldn't say I trained them! I put them on the straight and narrow.

Tansey: One of the things that intrigues me: you talked about the tea room and where ‘that Professor who works with Clive’. Has that always been a feature, of the technicians and the academics together?

Hood: No, no, it hasn't. At one time this particular room here was called the common room and this was where academics had their tea at five o'clock. There was no integration at those times. Oh God, we've got to go back a few years now. We're talking about 30, 35 years ago.

Tansey: Why did it change? Was there a policy of integration? Did somebody say this was all wrong? Were there any strong feelings in the department?

Hood: I don't know actually the reason why it was changed. When I first came here it was definitely touching the forelock where a technician was concerned; and as regards calling some academic by a first name, that was just unheard of.

Tansey: When do you think things started to change?

Hood: Well, it took me a long while to get out of the habit. I used to call Rushton Sir when he came in first thing in the morning, but I didn't dwell on it too often. But I never used to call him Willy or anything like that. I think I personally didn't change until I came back from America, because in America it was all first names whether you were a pleb or an academic. And Fergus insisted that I called him Fergus when I came back and started with him. So [it was] from there on. There's still certain people I don't call by their first names. I think it's probably respect more than anything. I mean, I wouldn't call Huxley Andy or anything like that. Although jokingly I once called Hodgkin Al because I wanted his parking space.

Tansey: What happened?

Hood: We went all over the country doing demonstrations. I did for Rushton quite a few, but I also did some for Fergus and we were invited to do a soirée once at the Royal Society. They are quite good. The thing that struck me most about that was the intelligence of the bright lads who came to the afternoon sessions. I mean, they grasped everything in a trice. But later, in the evening, when you give it to the eminent scientists, they seemed to not have a clue. I had to drive up to Carlton House in the old van with all the equipment and Alan Hodgkin was President of the Royal Society at the time and I knew he wasn't going up that day, and you know what parking's like in London. So I drove up in this old banger with the equipment and drove straight into Sir Alan's parking lot—the President's spot. This little chap came running out, peaked cap, you know, so I wound the window down and nonchalantly said, ‘Al said—oh, sorry, Sir Alan, said—that I could use his parking lot today, because he's not coming in.’ So this little chap with the peaked hat was all over me, he carried all the equipment in, so I was made for that day!

Tansey: Deserved elevation, Clive!
